# Heat-Induced Interactions between Whey Protein and Inulin and Changes in Physicochemical and Antioxidative Properties of the Complexes

**DOI:** 10.3390/ijms20174089

**Published:** 2019-08-21

**Authors:** Cuina Wang, Hao Wang, Xiaomeng Sun, Yuxue Sun, Mingruo Guo

**Affiliations:** 1 Department of Food Science, College of Food Science and Engineering, Jilin University, Changchun 130062, China; 2 Department of Food Science, College of Food Science, Northeast Agriculture University, Harbin 150030, China; 3 Department of Nutrition and Food Sciences, College of Agriculture and Life Sciences, University of Vermont, Burlington, Vermont, VT 05405, USA

**Keywords:** whey protein, inulin, dry-heating, physicochemical properties, antioxidative properties

## Abstract

Whey protein and inulin at various weight ratios were dry heated at 60 °C for 5 days under relative humidity of 63%. The heated mixtures were found to have significant changes in browning intensity and zeta-potential compared to untreated mixture. Heated samples showed significantly lower surface hydrophobicity than untreated mixtures. Compared with untreated samples, dry-heated samples showed significantly higher 2,2-Diphenyl-1-Picrylhydrazyl (DPPH) scavenging ability with whey protein to inulin mass ratios of 1:2 and 1:3 and significantly higher 2,2′-Azinobis(2-Ethylbenzothiazoline-6-Sulfonate) (ABTS) scavenging abilities and oxygen radical absorbance capacity (ORAC) at all weight ratios. Dry heat-induced interactions between whey protein and inulin was confirmed by changes in Sodium Dodecyl Sulfate Polyacrylamide Gel Electrophoresis (SDS-PAGE) protein profile, Fourier Transform Infrared Spectroscopy (FT-IR) and Far-ultraviolet Circular Dichroism (Far-UV CD) spectra. Dry heating caused physicochemical and structural changes of whey protein and therefore the complexes can be used to improve the antioxidative properties of the mixture under certain conditions.

## 1. Introduction

Proteins are important ingredients in the food industry due to their excellent nutritional properties and functionality. Attempts have been made to further improve their physicochemical and functional properties by various methods. Maillard reaction (MR) is a condensation reaction used to create new ingredients in protein samples by conjugation with sugar. It usually happens between reducing carbohydrates and proteins or amino acids during thermal treatments without introducing any toxic chemical products. Based on the state of the reaction system, MR can be categorized into types of dry-heating and wet-heating processes [1]. Dry heating process often happens in systems where materials are in dry state, and this kind of reaction often needs a long time and controlled conditions. Dry-heating method is reported to have relatively high reaction efficiency and can be used to improve the functionality of proteins [1].

Whey proteins (WP), the by-products of cheese making, have been extensively studied due to the high nutritional value and versatile functionality. Whey proteins include β-lactoglobulin (β-LG), α-lactalbumin (α-LA), bovine serum albumin (BSA), immunoglobulins, and other minor proteins [2]. ε-Amino groups of lysine and N-terminal amino group in proteins are the available reaction sites for MR in presence of reducing sugar. β-LG molecule contains 16 potential reactive amino groups (a terminal–NH_2_ and 15 lysine) while α-LA has 13 reactive amino groups (a terminal–NH_2_ and 12 lysine). Therefore, whey proteins have been extensively studied as Maillard reaction model substance. Numerous studies have been focused on changes in the physicochemical properties, solubility, emulsifying ability of whey proteins by covalent coupling with saccharides such as dextran [3], maltodextrin [4], pectin [5], and so on via dry heating method. In addition, MR has also been reported to improve antioxidant activity of whey proteins such as conjugating with xylose [6] and D-allose [7].

Inulin is usually a mixture of oligosaccharidic and polysaccharidic chains with β-D fructose or α-D glucose as terminal unit. Due to its reducing activity, inulin has been studied as substrate of Maillard reaction [8]. A mixture of fructose and inulin was used to improve the viscosity of caseinate by dry-heating at 60 °C at controlled water activities for 48 h [9]. Inulin was used to improve the radical scavenging activity and emulsifying properties of casein, ovomucoid, albumin and lysozyme by heating at 60 °C for 3 days, under a relative humidity of 79% [10]. Inulin and whey protein mixture was used to improve the DPPH scavenging activity of beetroot juice powders produced by spray drying [11].

Initial protein/polysaccharide ratio is an important factor in the Maillard reaction of whey proteins and polysaccharide [12]. Appropriate protein/polysaccharide weight ratio can result in high reaction speed and yield, and low side effects [13]; affect the binding ratio of whey protein [14]; and influence the functionality of the resultant glycated whey protein such as solubility, heat stability, and emulsifying properties.

This study aims to investigate changes in the physicochemical and antioxidative properties of whey protein and inulin mixtures (protein/polysaccharide mass ratios of 3:1, 2:1, 1:1 1:2 and 1:3) following controlled dry-heating.

## 2. Results and Discussion

Controlled conditions at 60 °C for 5 days under the relative humidity of 63% for the reaction were selected in the present study. In the condition of the solid phase at 60 °C at neutral environment, there should be a moderate reaction rate and no obvious side reactions [15]. A low temperature of 60 °C (below the denaturation temperature of whey protein) was also selected to minimize the loss in protein quality [16]. The relative humidity of 63% fell into the range between 50–80%, which was the optimum water activity for Maillard reaction [17].

### 2.1. Changes in Browning Intensity of Whey Protein and Inulin Mixture after Heating

**Figure 1:**
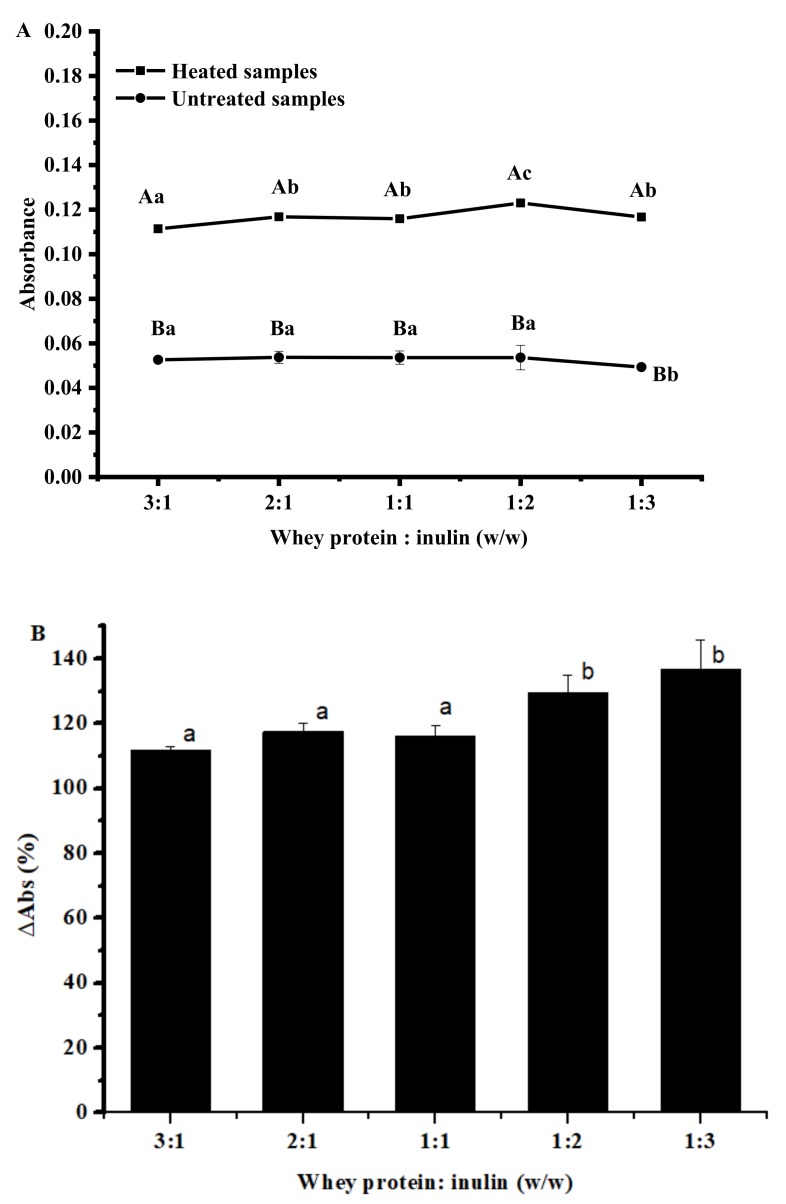
Browning intensity of heated and untreated whey protein and inulin mixtures (**A**) and changes in browning intensity of whey protein and inulin after heating (**B**). Different lowercase letters denote significant difference between samples at different ratios; Different uppercase letters denote significant difference between untreated and dry-heated samples at same whey protein and inulin ratio. Error bars are ± SD of the means.

Maillard reaction may produce colored reaction products, which depends on the stage of the reaction, pH, temperature, water activity, and so on [6]. At the initial stage, a colorless Schiff base is produced while brown pigments called melanoidins appear at final stages and they can be colorimetrically measured at 420 nm [18]. Brown color development of all whey protein: inulin ratio samples was evaluated and the results are shown in Figure 1. Figure 1A clearly showed that samples after dry-heating had significantly higher absorbance values than untreated samples, indicating an increased browning intensity. Similar results were reported by others on egg-white protein [10]. The analysis of the changes in absorbance (Figure 1B) showed that variable amount of later-stage MR products were produced at the variation of ratio of whey protein to inulin. In particular, samples at whey protein/inulin ratios of 1:2 and 1:3 showed greater changes than samples at ratios of 3:1, 2:1 and 1:1, and there was no significant difference between these two samples. Inulin has one reactive group (reducing end) binding to the amino group of the protein. The slight difference between these two ratios may be attributed to the number of inulin attached to whey protein and the reaction yield, which may be dependent on the protein conformation and polysaccharide steric hindrance [19].

### 2.2. Changes in Free Amino Groups Content of Whey Protein and Inulin Mixture after Heating

**Figure 2:**
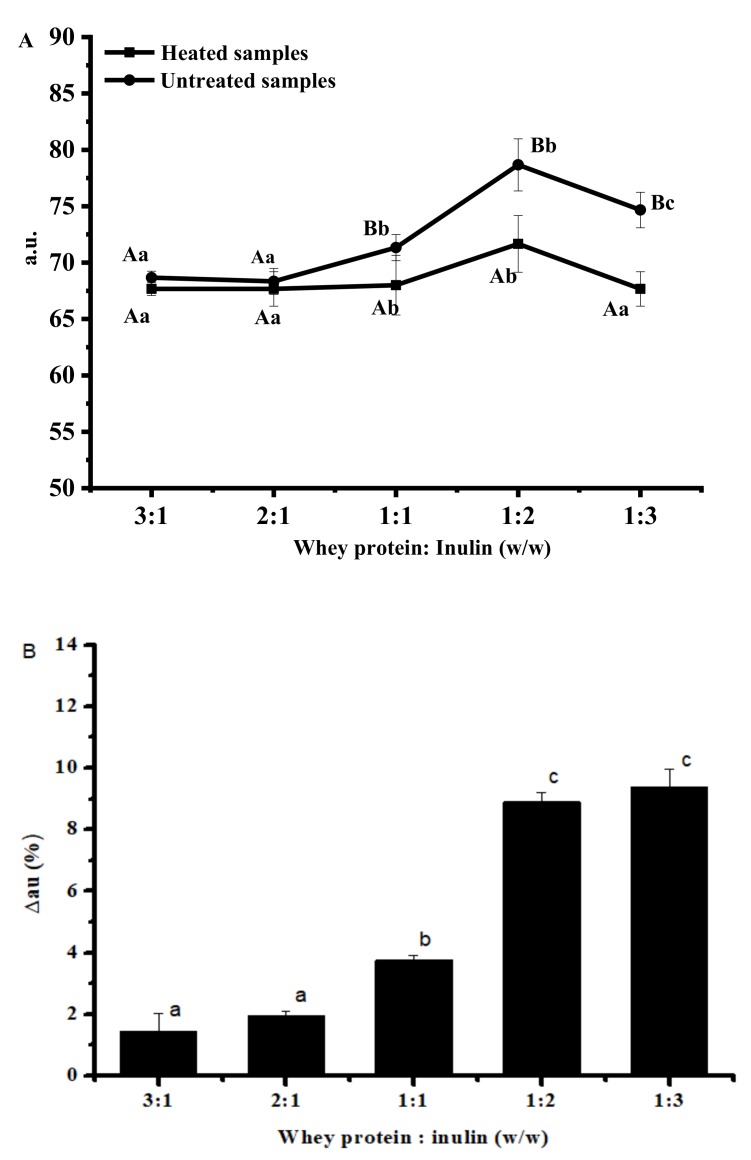
Free amino acid content (expressed by fluorescence intensity) of heated and untreated whey protein and inulin mixtures (**A**) and changes in fluorescence intensity of whey protein and inulin mixture after heating (**B**). Different lowercase letters denote significant difference between samples at different ratios; Different uppercase letters denote significant difference between untreated and dry-heated samples at same whey protein and inulin ratio. Error bars are ± SD of the means.

1,2-Phthalic dicarboxaldehyde (OPA) can react with the amino group of amino acid and form a quantifiable fluorescent compound, which can be used to measure the amino acids available for reactions. Free amino groups content of whey protein in the untreated and dry-heated mixture was determined and the results are shown in Figure 2. Compared with untreated mixtures, dry-heated samples at whey protein and inulin ratios of 1:1, 1:2 and 1:3 showed significantly decreased fluorescence intensity (Figure 2A), indicating the marked loss of free amino acid content in proteins after dry-heating. Changes of fluorescence intensity (Figure 2B) revealed that samples at ratios of 1:1, 1:2 and 1:3 showed significantly higher changes than other samples and there was no significant difference between samples at ratios of 1:2 and 1:3. The degree of reduced fluorescence intensity increased with the increasing whey protein to inulin ratio, indicating that the reaction between whey protein and inulin occurred much faster at higher ratios. When the ratio of whey protein to inulin increased to 1:2, it reached a reduction of about 9%. When the ratio was further increased to 1:3, there was no significant increase in fluorescence intensity, which may be due to the fact that the number of sugar was limited by the polysaccharide steric hindrance [19]. Compared with other saccharides conjugated to whey protein, it seemed that the decreased degree was much lower than those of dextran (30–35%) [3] and ribose (66%) [20]. The lower reactivity of inulin might be the result from a steric hindrance effects [21].

### 2.3. Changes in Zeta-Potential of Whey Protein and Inulin Mixture after Heating

**Figure 3:**
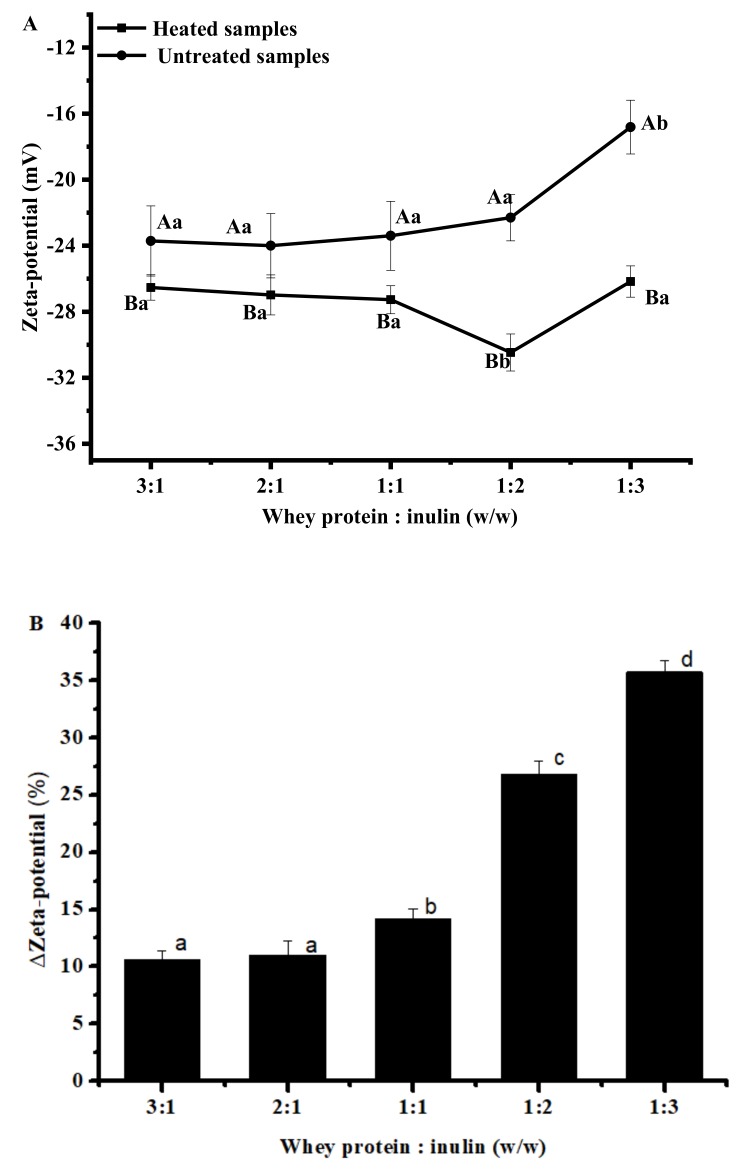
Zeta-potential of heated and untreated whey protein and inulin mixtures (**A**) and changes in zeta-potential of whey protein and inulin mixture after heating (**B**). Different lowercase letters denote significant difference between samples at different ratios; Different uppercase letters denote significant difference between untreated and dry-heated samples at same whey protein and inulin ratio. Error bars are ± SD of the means.

Maximizing the repulsive forces between particles to keep each particle discrete and prevent them from agglomerating is preferred for most food applications. Surface forces at the interface of the particle and the liquid can be measured by zeta-potential and the magnitude indicates the potential stability of the system. Zeta-potential was determined for all samples and the results are shown in Figure 3. Compared with respective untreated mixtures, all samples showed an increase in negative zeta potential values in the range of −26 to −31 mV (Figure 3A), indicating rather stable systems. Similar results were reported by others who found a significantly decreased zeta-potential values when pectin was dry-heated with whey protein at a mass ratios of 3:1, 2:1 and 1:1 [5]. As shown in Figure 3B, the zeta-potential changes between dry-heated samples and respective untreated samples varied, with samples at ratio of 1:3 exhibiting the greatest change (35.73 ± 0.95%). It should be noticed that although the greatest change was observed at samples with whey protein and inulin ratio of 1:3, the most stable mixture after dry heating occurred at ratio of 1:2 indicated by the highest negative zeta potential value.

### 2.4. Changes in Surface Hydrophobicity of Whey Protein and Inulin Mixture after Heating

**Figure 4:**
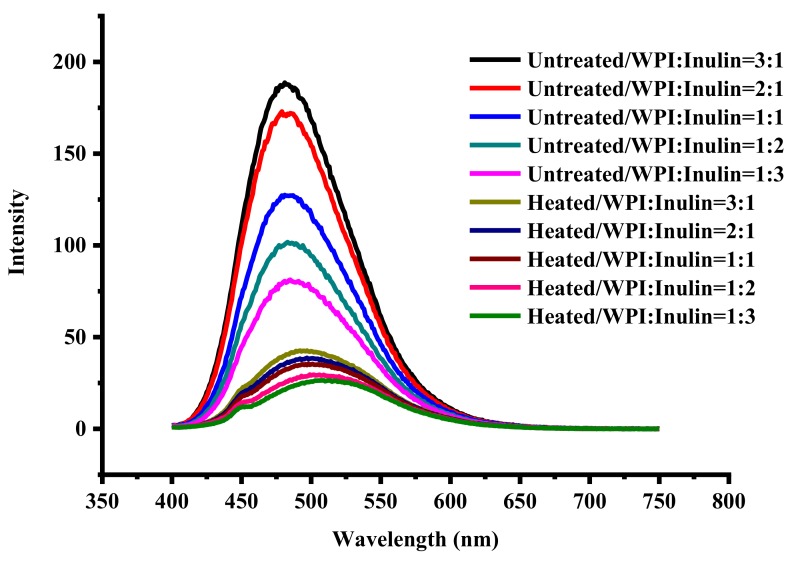
Surface hydrophobicity of whey protein and inulin mixture before and after dry heating.

Whey proteins have been extensively used in the area for fabrication of nutrient delivery. However, the high surface hydrophobicity often led to destabilization of the delivery system [22]. Polysaccharides are hydrophilic macromolecules and can be used to conjugate with protein to improve the stability [23]. All samples are measured for surface hydrophobicity and the results are shown in Figure 4. The mixture of whey protein and inulin had lower fluorescence intensity after dry-heating compared with controls, suggesting that the surface hydrophobicity of whey protein could be suppressed by incorporating inulin into the polypeptide chains. Similar results have been reported that the surface hydrophobicity of whey protein could be reduced by conjugating with dextran [3]. The decreased surface hydrophobicity may be due to the shielding effect of the bound polysaccharide chain [24]. It can be also observed that the fluorescence emission maximum (λ_max_) of mixtures moved to a higher wavelength (red shift) after dry-heating. The wavelength of maximum fluorescence intensity for untreated samples were 481–485 nm while that for the dry-heated samples moved to 497–500 nm. 8-Anilino-1-naphtalene sulfonic acid (ANS) probes were used to measure the aromatic hydrophobicity of proteins. The red shift may be a consequence of the conformation changes in whey proteins which contains a high proportion of hydrophobic amino acid side chains due to the dry-heating induced interaction between whey protein and inulin [25]. Compared with untreated samples, 77.38%, 77.52%, 72.17%, 70.95% and 67.55% reductions in the maximum fluorescence intensity were noted for samples with ratios from 1:3 to 3:1, respectively, indicating that less binding sites available for fluorescence probe ANS may be buried with increasing ratio due to the interaction between inulin and whey protein [26].

### 2.5. Changes in Antioxidative Properties of Whey Protein and Inulin Mixture after Heating

#### 2.5.1. Changes in 2,2-Diphenyl-1-Picrylhydrazyl (DPPH) Scavenging Ability

Table S1 revealed that percentage of inhibition was proportional to the concentration of whey protein-inulin mixture used. Dry-heated samples showed significantly lower DPPH scavenging capacity in comparison with untreated samples at the mass ratios of 3:1, 2:1 and 1:1, suggesting the lower electron-donating ability. The results indicated that antioxidant groups in whey proteins might be masked by heating at lower inulin levels. It was reported that whey protein may undergo denaturation and aggregation during heating even in dry state [27]. For samples where the protein portions were higher, a larger part of whey protein may participate into thermally induced aggregation, and our previous study showed that denaturation and aggregation of whey protein would result in decreased DPPH scavenging capacity. Other researchers found that flour protein showed reduction in electron transfer capacity after heating processing [28]. The author attributed the reduction to the isolating of relevant amino acids caused by structural changes in protein.

**Figure 5:**
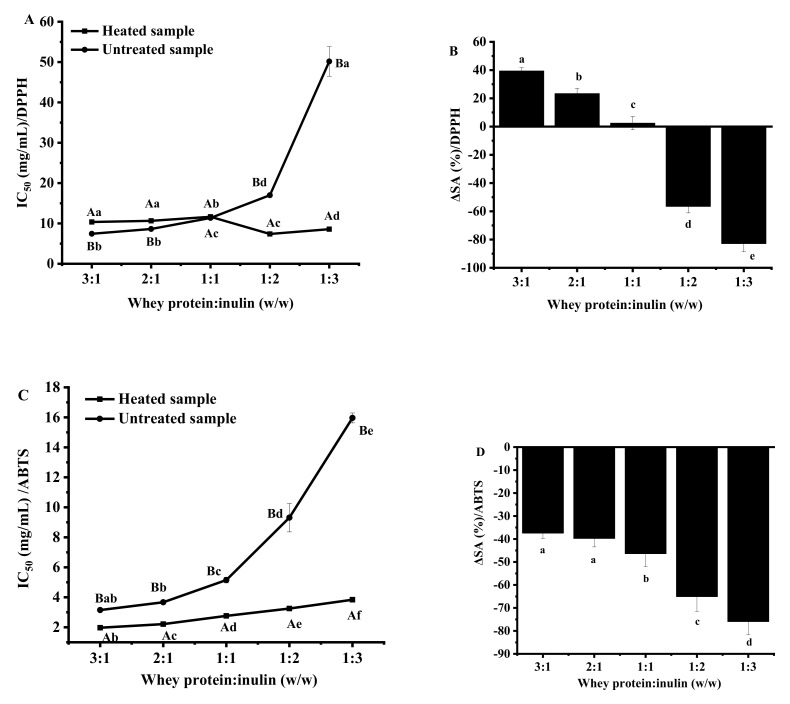
Changes in IC_50_ values of 2,2-diphenyl-1-picrylhydrazyl (DPPH) (**A** and **B**) and 2,2′-azinobis(2-ethylbenzothiazoline-6-sulfonate) (ABTS) (**C**,**D**) radical scavenging activities of whey protein and inulin mixture after dry heating. Different lowercase letters denote significant difference in IC_50_ values of DPPH or ABTS scavenging activities between samples with different whey protein and inulin ratios for untreated or dry-heated samples; Different uppercase letters denote significant difference in IC_50_ values of DPPH or ABTS scavenging activities between untreated and dry-heated samples at same whey protein and inulin ratio.

Significantly higher antioxidant capacities for dried samples were observed when inulin proportion increased to the ratios of 1:2 and 1:3. Accordingly, as shown in Figure 5A, samples at the ratios of 1:2 and 1:3 showed significantly lower IC_50_ values (7.38 ± 0.33 and 8.57 ± 0.29 mg/mL) than the respective untreated samples (16.99 ± 0.51 and 50.15 ± 3.65 mg/mL). The increased amplitude for samples at 1:2 and 1:3 were 56.56 ± 4.56% and 82.91 ± 5.78%, which were significantly higher than those of other samples (Figure 5B). Numerous studies have shown the good DPPH free radical scavenging capacity of Maillard reaction products developed by the reaction between sugar and protein. Glycosylated whey protein may provide hydrogen and form stable DPPH-H molecule, and thus scavenging the DPPH radical [29]. Improved DPPH radical scavenging activity of dry-heated mixture has also been reported for porcine plasma protein-glucose model system [30]. It was reported that intermediate and final Maillard reaction products (brown pigments) can act as hydrogen donors which contribute to DPPH radical scavenging capacity [28,31]. That may be the reason why samples at ratios of 1:2 and 1:3 with the greatest browning intensity changes had significantly higher DPPH radical scavenging capacity than other samples.

#### 2.5.2. Changes in 2,2′-Azinobis(2-Ethylbenzothiazoline-6-Sulfonate) (ABTS) Scavenging Ability

ABTS is peroxyl radical and is able to quench oxidant species by electron transfer [32]. Both untreated and dry-heated mixtures were determined for ABTS scavenging ability and the results are shown in Table S1. Obviously, dry-heating promoted the ABTS radical scavenging ability of the mixtures significantly when compared to untreated samples, *p* < 0.05. For both mixtures, scavenging ability decreased when whey protein and inulin ratios varied from 3:1 to 1:3, indicating the lower hydrogen-donating ability and less potency to react with free radicals. Calculated IC_50_ values for ABTS scavenging ability of all the samples are shown in Figure 5C. Heating significantly decreased the IC_50_ values by 37.46%, 39.78%, 46.41%, 65.09% and 75.94% for samples at whey protein and inulin ratios of 3:1, 2:1, 1:1, 1:2 and 1:3 (Figure 5D), respectively. Stronger ABTS-scavenging activity was also reported for mixture of α-lactalbumin and sugar reacted by dry-heating [7].

#### 2.5.3. Changes in Oxygen Radical Absorbance Capacity (ORAC) Values

**Figure 6:**
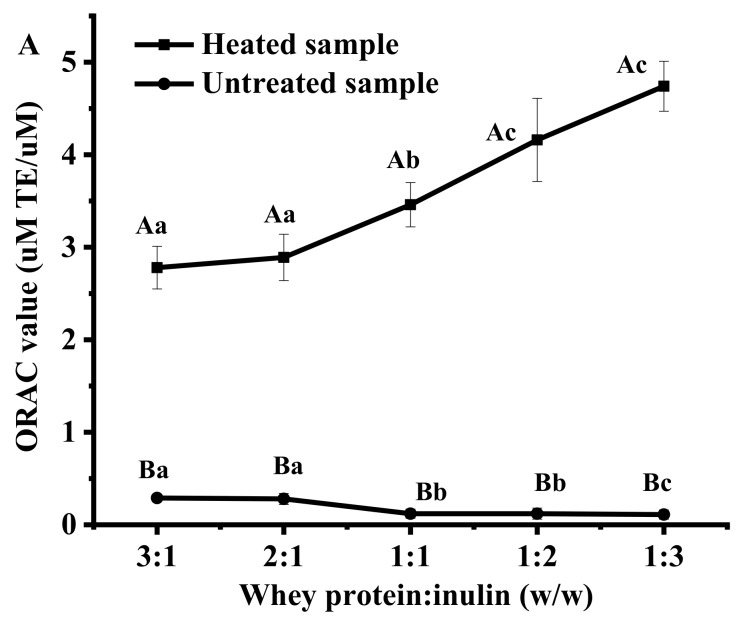

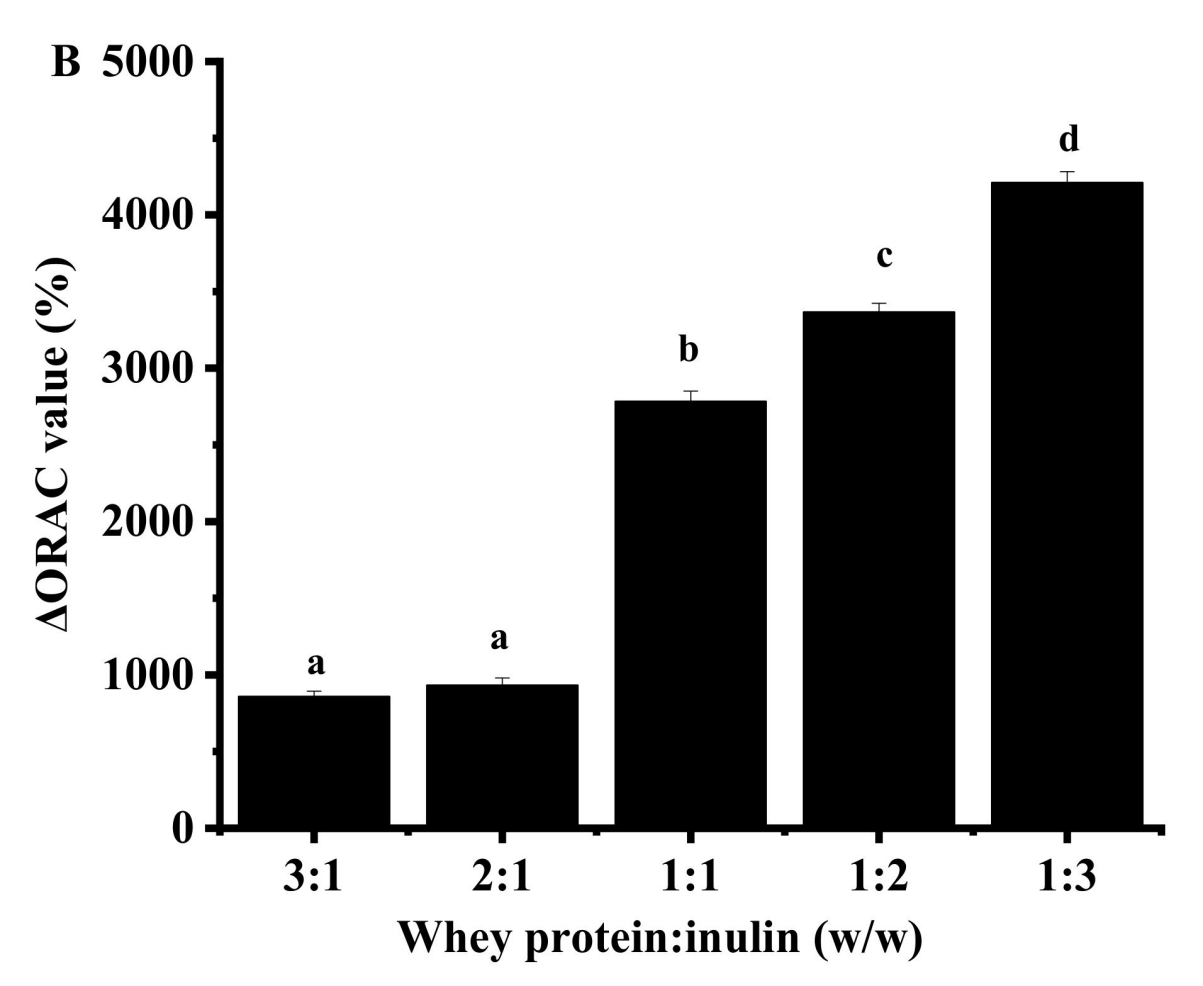
Oxygen radical absorbance capacity (ORAC) values (µM TE/µM) of heated and untreated whey protein and inulin mixtures (**A**) and changes in ORAC value of whey protein and inulin mixture after heating (**B**). Different lowercase letters denote significant difference in ORAC values between samples with different whey protein and inulin ratios for untreated or dry-heated samples; Different uppercase letters denote significant difference in ORAC values between untreated and dry-heated samples at same whey protein and inulin ratio.

Figure 6 shows the changes in ORAC values of whey protein and inulin mixtures after dry-heating. ORAC values of untreated samples decreased with inulin level increased, while the trend for heated samples was opposite. Compared with untreated mixtures, the heated samples showed significantly higher ORAC values. Similar results were reported in a previous study [28], where it was reported that there was an increase in the ORAC values of cricket flour protein in the presence of fructose after heating. Samples at whey protein and inulin ratios of 1:1 to 1:3 showed significantly greater change than samples at ratios of 3:1 and 2:1, indicating the changes in oxygen radical absorbance capacity.

### 2.6. Changes in Protein Profile Composition of Whey Protein and Inulin Mixture after Heating

**Figure 7:**
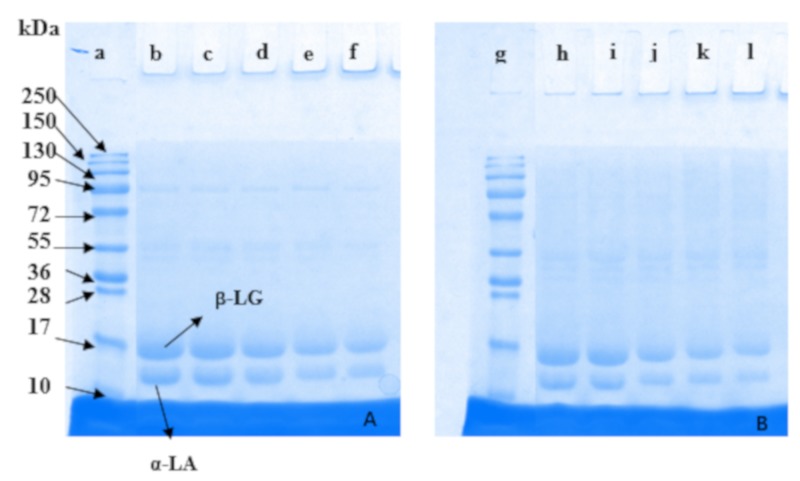
Protein profile of whey protein and inulin mixture before and after dry heating. (**A**) is untreated mixture and (**B**) is dry-heated mixture. a and g are Marker; b–f and h–l are samples at the ratios of whey protein to inulin of 3:1 to 1:3.

Mixtures were analyzed by comparing the electrophoretic pattern of the untreated and dry-heated protein samples in presence of inulin and the results are shown in Figure 7. The intensity of samples decreased with decreasing level of protein (Figure 7A,B). Compared with untreated samples, corresponding mixtures after dry-heating became slightly shallow for β-LG and α-LA and increased smearing at section of high molecular weight, indicating that whey proteins have been involved in interaction with inulin and new molecules may be produced. Samples at ratios from 1:1 to 1:3 showed decreased intensity to a greater extent compared with other samples. The results were consistent with those for browning intensity and free amino acid content. Similar results were reported for soy protein-dextran conjugates [33] where bands for soy protein became shallow and band with high molecular weight appeared.

### 2.7. Changes in Structures of Whey Protein and Inulin Mixture after Heating

**Figure 8:**
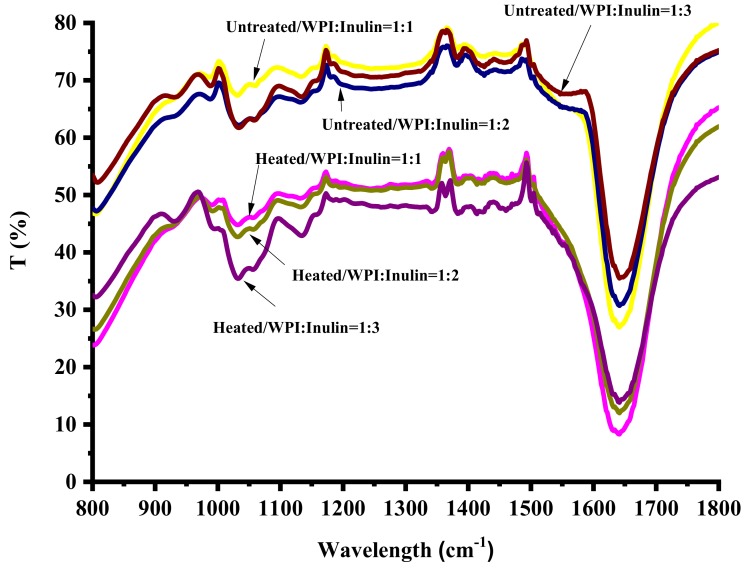
FT-IR spectra of whey protein and inulin mixture before and after dry heating.

**Figure 9:**
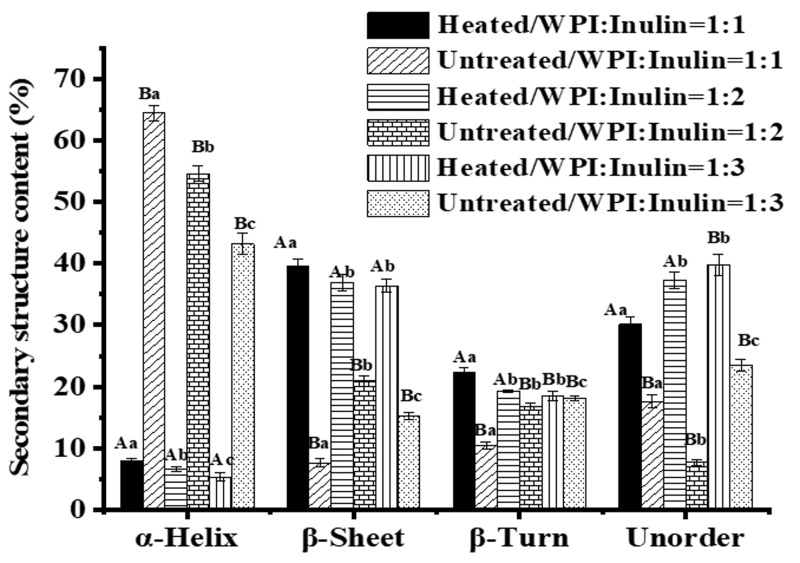
Secondary structures content (%) of whey protein in presence of inulin before and after dry heating. Different lowercase letters denote significant difference between samples with different whey protein and inulin ratios for untreated or dry-heated samples; Different uppercase letters denote significant difference between untreated and dry-heated samples at same whey protein and inulin ratio.

Maillard reaction causes changes in structure of whey protein due to formation of protein-sugar conjugates [34]. Fourier Transform infrared spectroscopy (FT-IR) and Circular Dichroism (CD) spectroscopies were used to determine changes in structure of whey protein in samples of at protein/inulin ratios of 1:1, 1:2 and 1:3 where relatively larger physicochemical and antioxidative properties changes were observed, and the results are shown in Figure 8 and Figure 9.

FT-IR spectroscopy is a useful technique for the study of protein-carbohydrate interactions systems [35]. As can be seen from Figure 8, absorption bands in the region of 1180–953 cm^−1^ were higher in dry-heated samples than untreated samples, indicating that there seemed to be a saccharide attached to whey protein [6]. Li et al. [21] also pointed out that the mixture of soy protein peptide and dextran after dry-heating showed strong absorption in the range of 1015 cm^−1^ and 1409 cm^−1^, which was mainly related to C-O and C-N stretching vibrations, indicating that dextran was attached to soy peptide. Frequency of approximate 1570 cm^−1^ was attributed to N-H plane bending [21]. Compared with untreated samples, dry-heated mixtures have a much smoother peak in the region of 1500–1600 cm^−1^. Maillard reaction is a condensation reaction of the carbonyl group of reducing sugar and available ɛ-amino groups of proteins. Therefore, -NH_2_ maybe lost after glycosylation. The bands at 1650 cm^−1^ was attributed to C=O association stretching which is amide I and 1570 cm^−1^ was attributed to N-H plane bending was amide II bands. Amide I and amide II bands are highly sensitive to the secondary structure of the polypeptide chain. Changes in the amide I and amide II indicated that the secondary structure of whey protein was modified by glycation with inulin [36].

Figure 9 shows the secondary structure content of whey protein and inulin mixture before and after heating calculated based on CONTIN method. Whey proteins are β-sheet rich proteins. However, whey protein mixed with inulin showed high level of α-helix content. The results suggested that the secondary structure of whey protein may be changed following the mixing and interaction. Similar results were reported for soy protein isolate [24] and peanut protein isolate [37] and the authors attributed this result to the interaction of protein and polysaccharide in solution. In addition, our previous study showed that inulin and whey protein may interact in solution through hydrogen bonding and hydrophobic interactions [38]. Whey protein after dry-heating in presence of inulin was reduced in α-helix and increased in β-sheet, β-turn and unordered structure. Decrease in α-helix was also reported for soy protein isolate conjugating with maltodextrin at 1:1 weight ratio after dry heating [24] and whey protein after glycating with xylose by heating in aqueous solutions [6]. Decreased α-helix and increased β-sheet and unorder was reported for ovalbumin after glycosylation with carboxymethyl cellulose via dry heating [13].

## 3. Materials and Methods

### 3.1. Materials

Whey protein isolate (WPI) was purchased from Fonterra Co-operative Group (Auckland, Netherlands). The percentage of protein is 92.3% based on dry weight. The whey proteins are composed of 69.2% β-lactoglobulin (β-LG), 14.2% α-lactalbumin (α-LA), 3.3% bovine serum albumin (BSA), and 2.1% immunoglobulin G (IgG). Inulin (93%, *w*/*w*) was provided by Zhangye Biological Technology Co., Ltd. (Gansu, China). Potassium bromide, 8-anilino-1-naphtalene sulfonic acid (ANS), and the reagents used for antioxidative properties assay were purchased from Sigma-Aldrich (St. Louis, MO, USA). SDS-PAGE gel preparation kit, SDS-PAGE sample loading buffer (5×), and coomassie blue fast staining solution were purchased from Beyotime Institute of Biotechnology (Nanjing, China). 1,2-Phthalic dicarboxaldehyde (OPA) was purchased from Solarbio (Beijing, China).

### 3.2. Dry-Heated Whey Protein and Inulin Mixture Preparation

Whey protein stock solution (20%, *w*/*v*) was prepared by dissolving whey protein powder in ultrapure water under stirring for 3 h and then hydrated at 4 °C overnight for complete dissolution. A correction for the protein purity (93.4%) of the powder was taken into account when calculating the amount of whey protein powder to be used. Whey protein and inulin at mass ratios of 3:1, 2:1, 1:1, 1:2 and 1:3 were mixed by dissolving inulin in appropriate diluted whey protein solution at constant total concentration of 10% (*w*/*v*). Sodium azide (0.02% final concentration, *w*/*v*) was added as a preservative. The mixed solutions were stored at 4 °C overnight for complete hydration. All the samples were adjusted to pH 7 with 2 N sodium hydroxide solution and then lyophilized using a freeze drier (ALPHA 1-2, CHRIST, Osterode, Germany). Dry-heated whey protein and inulin mixtures were prepared by incubating all the dried powder at 60 °C for 5 days, under the relative humidity of 63% in a desiccator containing a saturated potassium iodide solution. After heating, all samples were dissolved with water to the original levels and stirred for at least 30 min for dissolution. Untreated mixtures of whey protein and inulin adjusted to pH 7 were set as controls.

### 3.3. Browning Intensity Measurements

Browning intensity of all samples was measured for absorbance at 420 nm using a microplate reader (Synergy HT, BioTek, Winooski, VT, USA).

### 3.4. Free Amino Acid Content Determination

Free amino acid content of all samples was determined according to previous study with some modifications [39]. 1,2-Phthalic dicarboxaldehyde (OPA) reagent was prepared by dissolving 200 mg OPA powder, mixed with 125 mL sodium tetraborate solution (pH 9.75, 0.1 mM), 0.5 mL β-mercaptoethanol, and 12.5 mL SDS solution (10%, *w*/*v*), and then diluted to 250 mL using ultrapure water. Thirty-milliliter of samples (10-fold dilution) was mixed with 1 mL OPA reagent, vortexed, and kept for 5 min in darkness at room temperature. The fluorescence intensity was recorded at excitation wavelength of 360 nm and emission wavelength of 460 nm with a Spectrofluorometer (RF-5301PC, Shimatzu UV, Tokyo, Japan).

### 3.5. Zeta-Potential Determination

All samples were diluted to 1% (*w*/*v*) using ultrapure water and then determined for zeta-potential using a Zetasizer model Nano-Z (Malvern Instruments, Worcestershire, UK). All measurements were performed in triplicate. Data were calculated based on Henry equation.

### 3.6. Surface Hydrophobicity Measurement

All samples were diluted to 0.005% (*w*/*v*) and then measured for surface hydrophobicity by determining 8-anilino-1-naphtalene sulfonic acid (ANS) fluorescence intensity with a Spectrofluorometer (RF-5301PC, Shimatzu UV, Tokyo, Japan). ANS probe (20 μL, 8 mM) was added into 4 mL samples and then kept in darkness for 15 min. All samples were recorded for emission spectra from 400 to 700 nm at an excitation wavelength of 390 nm with slit width of 5 nm for excitation spectra and 3 nm for emission spectra at room temperature.

### 3.7. Antioxidative Properties Assay

#### 3.7.1. 2,2-Diphenyl-1-Picrylhydrazyl (DPPH) and 2,2′-Azinobis(2-Ethylbenzothiazoline-6-Sulfonate) (ABTS) Radical Scavenging Abilities

Scavenging activities of all samples on DPPH and ABTS radical were analyzed according to previous study [40]. Briefly, 150 μL samples at different concentrations (1–10 mg/g) were mixed with equivalent volume of DPPH solution (0.2 mM) under stirring, and then kept in darkness for 30 min at room temperature. The absorbance of mixtures was read at 517 nm by a microplate reader (Synergy HT, BioTek, Winooski, VT, USA). For ABTS scavenging ability analysis, 50 μL sample at different concentrations were mixed with 100 μL ABTS solution and then kept resting for 1 h at room temperature. All samples were recorded for absorbance at 734 nm. The scavenging abilities (SA) of samples were calculated using the following equation:

(1) $$SA\left(\%\right)=\left[1-\left(A_{s}-A_{B}\right)/A_{d}\right]\times 100$$

where A_B_, A_S_, and A_d_ represent the absorbance of the blank, DPPH/ABTS with samples and DPPH/ABTS solutions, respectively.

All samples were analyzed for half inhibition concentration (IC_50_, mg/mL) using regression equation by plotting linear plot of percent scavenging abilities VS. tested sample concentrations.

##### 3.7.2. Oxygen Radical Absorbance Capacity (ORAC)

Oxygen radical absorbance capacity of all samples were conducted according to previous study [41] with some modification. Briefly, 20 μL samples (0.05 mg/mL) or antioxidant (10 μM Trolox) were mixed with 120 μL fluorescein sodium (final concentration of 70 nM) in black 96-well microplates and incubated at 37 °C for 2 min in 75 mM phosphate buffer (pH 7.4), and then 60 μL 2,2′-azobis (2-methylpropionamidine) dihydrochloride (AAPH) at final concentration of 12 mM was added to start reaction. The plate was read for fluorescence every 2 min for 240 min at excitation wavelength of 485 nm and emission wavelength of 520 nm using a microplate reader (Synergy HT, BioTek, Winooski, VT, USA). Final ORAC values for samples were expressed as µmol Trolox equivalent/mg of protein (µM TE/µM).

### 3.8. Sodium Dodecyl Sulfate Polyacrylamide Gel Electrophoresis (SDS-PAGE)

Sodium dodecyl sulfate polyacrylamide gel electrophoresis (SDS-PAGE) of all samples was performed using a Mini-Protein Tetra Electrophoresis System (BioRad Laboratories, Hercules, CA, USA) with 12% acrylamide separating gel and 5% acrylamide stacking gel. Samples (7 μL, 8 mg/mL) diluted with SDS-buffer were loaded into each well. The separating gel was conducted at a constant voltage of 120 V and the stacking gel was conducted at a constant voltage of 80 V. After electrophoresis, the gel was stained for protein by Coomassie blue R250 for 30 min. Then the gel was destained by ultrapure water and then observed by Image Lab™ Software, Version 4.0 (Bio-Rad Gel Doc XR+, Bio-Rad Laboratories, Hercules, CA, USA).

### 3.9. Fourier Transform Infrared (FT-IR) and Far-ultraviolet Circular Dichroism (Far-UV CD) Spectroscopies

The FT-IR spectra of samples between 800 and 1800 cm^−1^ were recorded using an IRPRESTIGE-2 FT-IR spectrometer (Shimadzu, Tokyo, Japan). Samples (40 μL) were dropped uniformly on a potassium bromide slide. Potassium bromide powder was dried in a muffle at 140 °C for at least 2 h, and then 200 mg was weighed and pressed into slide. Blank potassium bromide was set as the control.

All samples were diluted to 0.1 mg/mL and then analyzed for changes in secondary structure by recording CD spectra from 190 to 250 nm using a CD spectropolarimeter (MOS-500, Bio-logic, Seyssinet-Pariset, France). Mean residue ellipticity ([θ], deg·cm^2^ dmol^−1^) was calculated using the following equation: [θ] = MWR × [θ]/10 × d × c, where θ corresponds to the observed ellipticity (deg); MRW is the mean residue weight, which was estimated to be 119 according to the main components of whey protein; d is the path length (0.1 cm); c is the protein concentration (mg/mL). The secondary structure contents of the samples were analyzed using DichroWeb online (http://dichroweb.cryst.bbk.ac.uk/html/home.shtml).

### 3.10. Statistical Analysis

Changes in physicochemical and antioxidative properties were calculated and expressed as percentage to values of untreated samples. All measurements were performed in triplicates for three trials and reported as mean ± standard deviation (SD). Statistical program SPSS Version 21 (SPSS Inc., Chicago, IL, USA) was used for statistical analyses. ANOVA was applied to analyze the differences between group data. Statistically significant difference was set at *p* = 0.05.

## 4. Conclusions

Heating whey protein and inulin dry mix at various ratios resulted in significant differences in physicochemical and antioxidant properties compared with untreated mixtures. The heated whey protein and inulin samples had higher browning intensity, absolute zeta-potential, and decreased surface hydrophobicity compared with control. Dry-heating improved the DPPH, ABTS, and AAPH radical scavenging activity at all ratios with the exception of the samples with whey protein/inulin ratios of 3:1 and 2:1 for DPPH radical scavenging activity. Structure change also occurred in whey protein molecules evidenced by shifting in FT-IR spectra and secondary structure content. The largest changes in antioxidantive properties at whey protein and inulin ratios of 1:1, 1:2 and 1:3 may be due to the largest changes in physicochemical properties and structure.

## Supplementary Materials

Supplementary materials can be found at https://www.mdpi.com/1422-0067/20/17/4089/s1.
