# Supplementary material for: Heat-Induced Interactions between Whey Protein and Inulin and Changes in Physicochemical and Antioxidative Properties of the Complexes
Source: Int J Mol Sci. 2019 Aug 21;20(17):4089. doi: 10.3390/ijms20174089 (PMC6747464; doi:10.3390/ijms20174089)
Supplement: Supplementary file 1 [file ijms-20-04089-s001.pdf]

Table 1 Changes in DPPH and ABTS scavenging abilities of whey protein and inulin mixture after dry heating

	Untreated sample				Dry-heated sample			
	1 mg/mL	2.5 mg/mL	5 mg/mL	10 mg/mL	1 mg/mL	2.5 mg/mL	5 mg/mL	10 mg/mL
**DPPH**								
WPI: inulin =3:1	15.48 ± 1.07^{Aa}	34.68 ± 1.14^{Aa}	44.91 ± 1.30^{Aa}	52.24 ± 3.52^{Aa}	14.64 ± 1.85^{Aa}	24.00 ± 2.35^{Ba}	40.83 ± 1.24^{Ba}	48.69 ± 2.89^{Ba}
WPI: inulin =2:1	11.27 ± 0.99^{Ab}	30.81 ± 0.98^{Ab}	43.30 ± 2.63^{Aa}	48.89 ± 1.59^{Ab}	13.72 ± 1.17^{Bbc}	24.94 ± 3.08^{Bab}	41.24 ± 2.32^{Ba}	49.69 ± 1.55^{Aa}
WPI: inulin =1:1	2.17 ± 0.37^{Ac}	22.92 ± 1.36^{Ac}	36.52 ± 2.29^{Ab}	45.38 ± 1.79^{Aa}	12.12 ± 1.05^{Bc}	25.85 ± 1.22^{Bbc}	36.19 ± 1.00^{Aa}	48.87 ± 1.78^{Ba}
WPI: inulin =1:2	2.01 ± 0.28^{Ac}	12.87 ± 0.68^{Ad}	29.35 ± 1.31^{Ac}	40.63 ± 2.03^{Ad}	12.39 ± 0.91^{Bc}	26.20 ± 0.97^{Bb}	41.34 ± 1.70^{Ba}	56.96 ± 1.69^{Bb}
WPI: inulin =1:3	2.68 ± 0.34^{Ac}	3.61 ± 0.29^{Ae}	20.15 ± 1.85^{Ad}	32.88 ± 2.41^{Ae}	12.87 ± 1.77^{Bc}	23.79 ± 0.78^{Bad}	39.05 ± 0.85^{Bb}	53.70 ± 1.68^{Bc}
**ABTS**								
WPI: inulin =3:1	14.47 ± 0.62^{Aa}	37.84 ± 1.59^{Aa}	70.65 ± 1.64^{Aa}	83.72 ± 3.07^{Aa}	30.60 ± 2.29^{Ba}	54.66 ± 1.18^{Ba}	76.83 ± 1.47^{Ba}	90.40 ± 0.38^{Ba}
WPI: inulin =2:1	13.84 ± 1.25^{Aa}	34.74 ± 2.53^{Ab}	64.78 ± 1.81^{Ab}	77.19 ± 1.11^{Ab}	29.05 ± 1.12^{Bb}	52.17 ± 1.66^{Bb}	74.24 ± 2.15^{Bb}	89.27 ± 0.47^{Bb}
WPI: inulin =1:1	5.17 ± 0.47^{Ab}	24.55 ± 1.24^{Ac}	53.05 ± 2.83^{Ac}	67.77 ± 1.87^{Ac}	23.73 ± 0.94^{Bc}	46.40 ± 0.89^{Bc}	67.03 ± 0.88^{Bc}	84.40 ± 1.14^{Bc}
WPI: inulin =1:2	1.81 ± 0.18^{Ac}	13.70 ± 1.20^{Ad}	39.57 ± 2.79^{Ad}	52.42 ± 3.11^{Ad}	19.30 ± 0.92^{Bd}	39.14 ± 1.10^{Bd}	62.83 ± 1.54^{Bd}	81.93 ± 1.80^{Bd}
WPI: inulin =1:3	1.02 ± 0.04^{Ad}	7.72 ± 0.78^{Ae}	31.41 ± 1.20^{Ae}	43.56 ± 1.29^{Ae}	19.26 ± 1.55^{Bd}	32.12 ± 1.40^{Be}	57.70 ± 2.43^{Be}	77.68 ± 1.26^{Be}

Different uppercase letters denote significant difference at $P < 0.05$ in DPPH or ABTS scavenging ability between native and dry-heated samples at same concentration and same whey protein and inulin ratio; Completely different lowercase letters denote significant difference at $P < 0.05$ in DPPH or ABTS scavenging ability between samples with different whey protein and inulin ratios at same concentration for native or dry-heated samples.
